# Home Range and Habitat Use of the Swan Goose (*Anser cygnoides* L. 1758) during Wintering in the Seocheon Tidal Flat, South Korea, Using GPS-Based Telemetry

**DOI:** 10.3390/ani12213048

**Published:** 2022-11-06

**Authors:** Sungbae Joo, Yu-Seong Choi, Sang-Yeon Lee

**Affiliations:** 1 Ecological Technology Research Team, National Institute of Ecology, Seocheon 33657, Korea; 2 National Migratory Bird Center, National Institute of Biological Resources, Incheon 22689, Korea; 3 Division of Ecological Survey Research, National Institute of Ecology, Seocheon 33657, Korea; 4 School of Biological Sciences and Biotechnology, Chonnam National University, Gwangju 61186, Korea

**Keywords:** *Anser cygnoides*, staging site, home range, habitat use, waterbirds

## Abstract

**Simple Summary:**

Due to the rapid environmental changes in the Seocheon Tidal Flat, South Korea, important staging and wintering sites of the vulnerable Far East Russin population of Swan Goose are threatened. To provide practical information for establishing protection strategies based on Swan Goose behavioral characteristics, we estimated core home range and habitat use patterns over time at the Seocheon Tidal Flat during wintering. Based on the GPS tracking data, the core home range and habitat use characteristics of the Swan Goose differed significantly between daytime and nighttime (Day: 59.9 km^2^, Night: 40.3 km^2^, on average, 100% MCP). In addition, our data indicated that Swan Goose has two important overnight resting areas in the Seocheon Tidal Flat, South Korea, namely the Janggu Bay and sand dune areas in Yubu Island.

**Abstract:**

The Seocheon Tidal Flat is an important staging and wintering site for the Far East Russian population of Swan Goose (*Anser cygnoides*) in the East Asian–Australasian Flyway. However, rapid environmental changes for tourism in this area can threaten the survival of this vulnerable population by hindering sufficient rest and wintering; therefore, establishing protection strategies based on Swan Goose behavioral characteristics is necessary. Here, we estimated Swan Goose core home ranges and habitat use based on GPS tracking data collected at the Seocheon Tidal Flat in South Korea from 2017–2018. The home range of Swan Geese was estimated to be an area from Yubu Island in the south to Janggu Bay in the north; however, the core home range and habitat use characteristics differed significantly between daytime and nighttime (Day: 59.9 km^2^, Night: 40.3 km^2^, on average, 100% MCP). During the day (08:00–18:00), Swan Geese mostly spent time resting or feeding on tidal flats, especially those around tidal channels or paddy fields near Janggu Bay, whereas they mostly rested on sand dunes near Yubu Island along with the mudflats at Janggu Bay at night. Our results provide practical information on the habitat use of wintering Swan Geese population over time and indicate that Yubu Island is an important resting place. Hence, these results can contribute to evaluating threats to Swan Geese and establishing management and protection strategies for the Seocheon Tidal Flat, a major wintering site for the Far East Russian population of Swan Geese.

## 1. Introduction

Many migratory birds depend on stopover sites where they must rest and refuel energy during long-distance migration [1]. The East Asian–Australasian Flyway (EAAF) is one of nine major flyways worldwide (www.eaaflyway.net, accessed on 27 September 2022). This flyway encompasses 22 countries and extends from Russia and Alaska to Southeast Asia, Australia, and New Zealand. The flyway’s highly productive coastal ecosystem (i.e., tidal flats and estuaries) is a repository of biodiversity and an important area used as a stopover and staging site for various migratory birds [2]. In particular, tidal mudflats of the Yellow Sea ecosystem (known as “*Getbol*”) represent the largest muddy tidal area worldwide [3]. This renowned ecosystem is a vital staging site for migratory waterbirds in the EAAF [4]. Furthermore, the Seocheon Tidal Flat in South Korea is also important for supporting various waterbirds including globally-threatened bird species [5,6]. However, many migratory birds are constantly threatened by human activity. The Yellow Sea ecosystem containing the Seocheon Tidal Flat is continuously being degraded, with over 1% of its tidal mudflats being destroyed annually by coastal development [7]. Several studies suggest that environmental changes in tidal flats along the Yellow Sea coast pose a serious threat to the survival of migratory waterbird populations [8,9]. 

The Swan Goose (*Anser cygnoides*) is a globally-threatened species categorized as “Vulnerable, VU” on the Red List of Threatened Species of the International Union for Conservation of Nature (IUCN) [10,11]. Many studies have reported a rapid population decline due to uncontrolled hunting, egg collection, anthropogenic disturbance, prolonged drought, and agricultural development. The Swan Goose is endemic to East Asia and is largely divided into populations breeding in Mongolia and northeastern China and the Amur River basin in Russia [10,11,12,13,14]. The global population has been estimated to be approximately 60,000–90,000 individuals, but the Far East Russian population is known to be less than 10% of the global population [10,11]. In addition, the Far East Russian population may have been genetically isolated from the Chinese and Mongolian populations [15]. Previous studies using neck-collar marking and telemetry tracking reported that the Far East Russian population commonly visits and uses two regions (the Han River Estuary and Seocheon Tidal Flat, South Korea) as wintering and stopover areas [13,16]. Nevertheless, population decline is a concern owing to the low survival rate of the swan goose, and land use changes by human activities in these areas may threaten wintering Swan Geese [12].

Spatial information for bird species is essential for establishing local ecosystem conservation strategies [12,17]. Quantitative threat assessment based on habitat range estimation and habitat use pattern would be preceded to establish conservation strategies for wintering Swan Goose populations. This study aimed to provide practical information for establishing protection strategies for the Swan Goose by estimating core home range and habitat use patterns over time during wintering at the Seocheon Tidal Flat through GPS tracking.

## 2. Materials and Methods

### 2.1. Study Area

**Figure 1:**
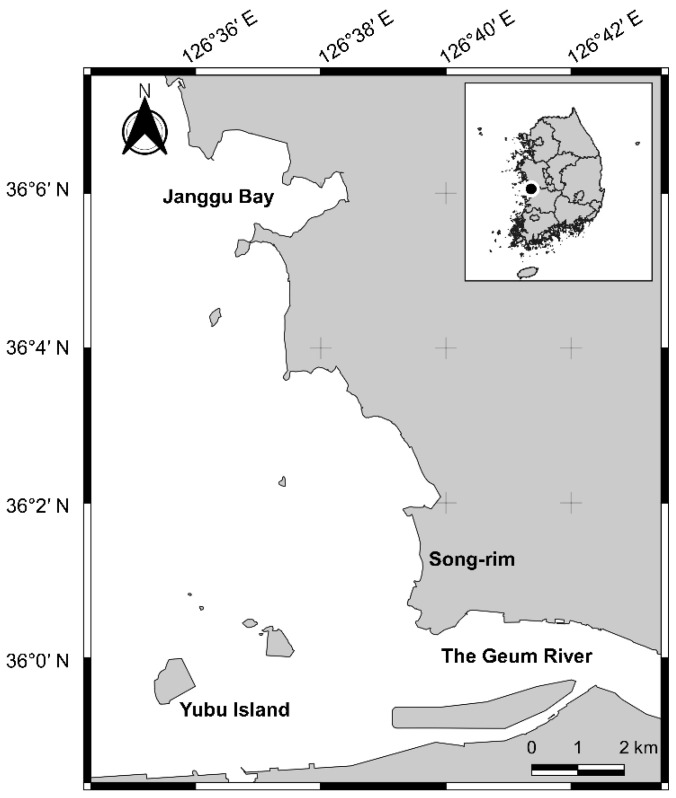
Map of the study area based on the GPS tracking dataset.

In this study, we focused on the Seocheon Tidal Flat in South Korea, where approximately 60 individual Swan Geese were wintered during 2014–2015 [13]. The study area includes a tidal flat, which is composed of a combination of sand and/or muddy sand flats, agricultural areas, and some islands, such as Yubu Island and Geumran Island, around the Geum River estuary (126°36′–126°43′ E; 35°8′–36°6′ N; Figure 1). The Seocheon Tidal Flat has been internationally designated as a Ramsar site, registered as a World Heritage List, and is protected by the Ministry of Oceans and Fisheries, Republic of Korea [18,19].

### 2.2. GPS Data Collection and Preprocessing

The swan goose is designated as a natural monument by the Cultural Heritage Administration of the Republic of Korea. The Cultural Heritage Administration approved all handling and capturing permission through the local government (Seocheon 2017-1 to Joo). We adhered to the Republic of Korea’s Wildlife Protection and Management Act and Institutional Research Ethics Regulations and Guidelines. We captured four Swan Geese (body weight range: 3420–4190 g) on 15 November 2017, accounting for about 9.5% of the total number of geese at the time of capture (Table 1). Upon capture, each individual was fitted with a neck-collar type GPS–WCDMA transmitter (Model WT-300, KoEco, Republic of Korea). Each transmitter weighed 27 g and did not exceed 1% of the bird’s weight to minimize behavioral constraints [20]. The individual was immediately released when the transmitter attachment was completed. We did not conduct additional capturing in order to reduce the negative effects of repeated capturing behavior on Swan Goose populations. The transmitter was programmed to collect position data on time, every two hours daily, and the acquired data were transmitted to the server twice daily. The GPS tracking dataset was recorded until 19 April 2018, but the GPS tracking dataset that could be acquired from the transmitter was limited to the period of stay at the Seocheon Tidal Flat in this study. Each individual was assigned a unique number (NIE1701 to NIE1704; NIE: National Institute of Ecology) and tracked until the signal was lost. For this study, the GPS tracking dataset acquired from the transmitter was limited to the period of stay at the Seocheon Tidal Flat. The individual NIE1703 left the study area within three days of marking; therefore it was excluded from the analysis (Table 1).

### 2.3. Home Range and Habitat Use Analysis

We estimated the home range areas of the Swan Goose at the Seocheon Tidal Flat using the R package ‘adehabitatHR’ and QGIS 3.24.2 [21,22,23]. The ranging areas were computed as 100% minimum convex polygons (MCP) and kernel density estimation (KDE; 90%, 70%, and 50%) using the least-squares cross-validation (LSCV) method. MCP is a method of estimating the home range by connecting the outermost coordinates; its disadvantage is the inclusion of regions not used in the home range [24]. KDE is a method of estimating the distribution characteristics of an object from the collected coordinates using a kernel function, where 90% of the probabilities are derived from the normal home range, and 50% are derived from the core home range [24,25]. We divided the GPS dataset into day and night based on the sunrise and sunset times of the Geumgang Estuary to compare the difference in home range between daytime and nighttime. GPS data recorded from 08:00 to 18:00 were classified as “Day,” and others (recorded from 20:00 to 06:00 of the next day) were classified as “Night.” Habitat use of Swan Geese was estimated based on land use types, where GPS location information was detected every two hours. Based on the results of previous studies, land use was categorized into four types: tidal flats, paddy fields, near shorelines, and others [13]. We compared the statistically estimated home range between daytime and nighttime using the non-parametric Mann-Whitney U-test.

## 3. Results

A total of 1572 location fixes were recorded when three Swan Goose individuals stayed on the Seocheon Tidal Flat (Table 1). The longest period of data was obtained from NIE1702 (99 nights), whereas the other two individuals had 28 nights of data. Two swan geese (NIE1701 and NIE1704) left the Seocheon Tidal Flat on 12 December 2017, and the other (NIE1702) left on 22 February 2018 (Table 1 and Table S1). 

**Figure 2:**
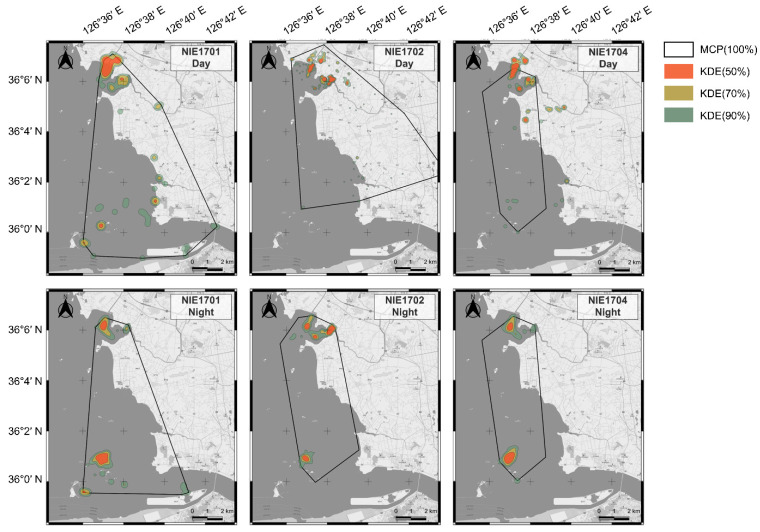
Home range estimation for Swan Geese using a minimum convex polygon (MCP) and kernel density estimation (KDE 50%, 70%, 90%) between day (08:00–18:00) and night (20:00–06:00).

The home range of the three Swan Geese included Yubu Island in the south and Janggu Bay in the north, around the Seocheon Tidal Flat (Figure 2). Each individual had similar home ranges and exhibited no statistically significant difference during the day and night (35.86–76.09 km^2^ in the day and 31.89–51.77 km^2^ at night, 100% MCP, Mann–Whitney *U* test, *Z* value: 1.0911, *p* = 0.4; Table 2). The home range calculated by LSCV-KDE was estimated from 0.4–7.06 km^2^ according to three different percentage levels (50%, 70%, and 90%). The average size of the home range (90% KDE) was 4.1 km^2^ (range: 2.33–7.06 km^2^), and the home range (50% KDE) was 0.7 km^2^ (range: 0.4–1.05 km^2^) during the day. At night, the average size of the home range (90% KDE) was 3.0 km^2^ (range: 2.44–3.84 km^2^), and the home range (50% KDE) was 0.6 km^2^ (range: 0.47–0.69 km^2^). NIE1701 exhibited the widest home range day and night compared to the other two individuals. However, the location of the core home range (50% KDE) demonstrated significant differences (Figure 2). During the day, the core home range (50% KDE) mainly included the Janggu Bay area (tidal flat and channel, paddy fields). However, at night, the core home range (50% KDE) included both sand dunes located in the northeastern part of Yubu Island and the Janggu Bay area (tidal flat and channel; Figure 2).

**Figure 3:**
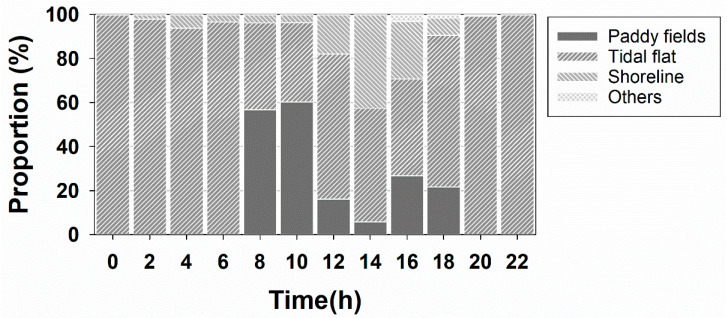
Proportion of habitat use for Swan Goose at two-hour intervals.

The proportion of the total time spent in specific habitats suggested that Swan Goose individuals spent the greatest proportion of time on tidal flats (74.4%) and less time in paddy fields (15.6%), shorelines (9.6%) and others (0.4%) (Figure 3). The relative proportions of time spent in the four habitats differed between day and night. During the daytime (08:00–18:00), Swan Geese mostly spent time on tidal flats (especially around tidal channels), coastal, and agricultural areas. At night, they mostly stayed in tidal flats, including sand dunes near Yubu Island. GPS tracking information was recorded around sand dunes northeast of Yubu Island and around tidal channels far from the shorelines in Janggu Bay. The proportion of time spent in paddy fields increased in the early morning (56.8%–60.2%; 08:00–10:00) and late afternoon (21.8%–26.7%; 16:00–18:00).

## 4. Discussion

Our results indicate that the core winter season home range of the Far East Russian population of Swan Goose is located in Janggu Bay and sand dunes near Yubu Island in the Seocheon Tidal Flat, South Korea (Day: 59.9 km^2^, Night: 40.3 km^2^, on average, 100% MCP). Based on the GPS tracking data, the core home range and habitat use characteristics of the Svan Goose differed significantly between daytime and nighttime. During the daytime, Swan Geese mostly spent time on tidal flats and agricultural areas near Janggu Bay for food and rest, and they mostly stayed in tidal flats, including sand dunes near Yubu Island, at night. Specifically, sand dunes near Yubu Island were estimated as the core home range area at night for the Swan Geese. 

Our results support previously published data and suggest that Swan Geese mainly used Janggu Bay in the Seocheon Tidal Flat after early wintering [13]. The core home range estimated in this study includes paddy fields near Janggu Bay, where harvest has been completed. Generally, the Swan Goose is known to feed on the underground parts (rhizomes or tubers) of aquatic plants; tuberous bulrush (*Bolboschoenus planiculmis*) is a popular food for Swan Geese [26]. Similar to other ducks and geese, the Swan Goose consumes energy for overwintering by grazing mainly on stems and tubers in the surface layer when the water level drops below the substrate surface [27,28]. Tuberous bulrush is widely distributed in the Song-rim coastal area and Janggu Bay in the Seocheon Tidal Flat. Thus, Swan Geese stayed and fed on favorite food sources in this area during the early winter [13,26,29]. However, after the early winter, the energy deficit is replenished by eating the grains left after harvest or roots of other plants like legumes and sedges in paddy fields [13]. Accordingly, the core home range of the Swan Goose during the daytime expands to paddy fields, which reflects the general behavioral patterns of herbivorous geese that typically spend most of their time grazing in daylight to achieve their energetic requirements [30,31]. In contrast, the core home range area was estimated mainly in tidal flats and channels away from the coastlines or sand dunes near Yubu Island at night. Furthermore, our GPS tracking discovered the importance of the sand dunes near Yubu Island as roosting sites for the Swan Goose, which may reduce the threat from predators. 

Identifying important sites on the flyway and ensuring adequate protection are essential factors for conserving various migratory waterbird populations [4,8]. In addition, the expansion of protected areas on migratory routes is recommended by the United Nations Convention on Biological Diversity (CBD) [32]. Establishing a protected area for specific bird species should be based on behavioral information, such as habitat use, roosting, and foraging, as many migratory birds depend on specific stopover sites in intertidal habitats (tidal flats) during migration [8,33]. For example, it may be an effective measure to artificially feed grains to birds that feed mainly in rice paddies or to provide shallow water habitats for species that feed mainly in rivers, such as Whooper Swan (*Cygnus cygnus* L. 1758) and Bean Goose (*Anser fabalis* Latham 1787) [5]. Similarly, various conservation policies can be established, such as artificially supplying grains in winter, leaving behind some grain after the harvest, and designating the paddy fields around Janggu Bay as key protection areas based on the information on the habitat use of Swan Geese. These policy measures may also positively affect other migratory waterbirds that share wintering areas [34,35]. Our results have a limitation in that the estimated home range in this study was derived from only one-year tracking results of three Swan Goose individuals. Therefore, it is necessary to derive a more accurate core home range through continuous tracking of habitat use information in the future. Nevertheless, the information on the core home range estimated in this study can be valuable for protecting the Far East Russian population of Swan Goose, which has a significantly small number and exhibits genetic differentiation compared with the Chinese and Mongolian populations [12,13,15].

South Korea’s protected area system is well managed, and efforts are being made to conserve migratory waterbirds and wetlands through various international activities [4,8]. Nevertheless, the degradation of tidal flats occurred overwhelmingly outside the protected area, and continuous anthropogenic disturbances, such as the construction of tourism facilities around the protected area, may adversely affect the temporal and spatial distribution of migratory waterbirds [4,5,8,36,37]. For stable wintering of Swan Geese in the Seocheon Tidal Flats, it is necessary to prepare management and protection measures according to habitat use characteristics. The estimated core home range information in this study may provide practical information for assessing the threat posed by human activities to the Seocheon Tidal Flat as a wintering site.

## 5. Conclusions

The current study determined the core home ranges and habitat use of Swan Geese during their stay at the Seocheon Tidal Flat. The core home range of Swan Geese was estimated to include Yubu Island in the south up to Janggu Bay in the north, around the Seocheon Tidal Flat during wintering. Our results indicate that paddy fields around Janggu Bay play an important role as a major feeding area for energy replenishment for Swan Geese after early winter. In addition, this study presented the importance of the sand dunes near Yubu Island as resting sites for the Swan Goose, which may reduce the threat of predators at night. Our findings offer practical information that can be used to develop management and protection strategies for migratory birds in the Seocheon Tidal Flat. Policy measures, such as designating paddy fields around Janggu Bay as key protection areas, artificially supplying grains, or leaving some grains after the harvest, can be considered for energy replenishment and wintering of Swan Geese.

## Figures and Tables

**Table 1:** Description of GPS tracking for three Swan Geese. The GPS tracking dataset was limited to the period of stay around the Seocheon Tidal Flat, and the individual (NIE1703) that left after attaching the tracker was excluded from this study.

Species	ID	Weight (kg)	First Date Tracked	Last Date Tracked	Day	Data Points
*Anser cygnoides*	NIE1701	4.19	15 November 2017	12 December 2017	28	272
	NIE1702	3.42	16 November 2017	22 February 2018	99	1030
	NIE1704	3.5	15 November 2017	12 December 2017	28	270

**Table 2:** Home range estimations for each Swan Goose individual.

Tracking ID	Time	MCP (km^2^)	KDE (km^2^)		
			50%	70%	90%
NIE1701	Day	76.09	1.05	2.62	7.06
	Night	51.77	0.69	1.55	3.84
NIE1702	Day	67.86	0.40	0.95	2.33
	Night	37.54	0.47	1.02	2.44
NIE1704	Day	35.86	0.60	1.34	2.91
	Night	31.89	0.53	1.09	2.58
Average (SE)	Day	59.9 (12.3)	0.7 (0.2)	1.6 (0.5)	4.1 (1.5)
	Night	40.3 (5.9)	0.6 (0.1)	1.2 (0.2)	3.0 (0.4)

MCP = Minimum Convex Polygon, KDE = Kernel Density Estimate, SE = Standard Error.

## Data Availability

The data used in this study were accessed from Movebank (movebank.org, study name “Swan Goose NIE 2017 South Korea,” study ID 2234937659).

## Supplementary Materials

The following are available online at https://www.mdpi.com/article/10.3390/ani12213048/s1, Table S1: The population change of Swan Goose wintering at the Seocheon Tidal Flat in South Korea during 2017–2018.
